# Characteristics of severe injuries among children and adolescents in a sub-national trauma registry in Saudi Arabia

**DOI:** 10.3389/fpubh.2025.1651372

**Published:** 2025-11-27

**Authors:** Meshal Alqahtani, Lubna A. Alnasser, Maram A. Aldosari, Yasir Almuzaini, Abdullah Alqahtani, Ibrahim Albabtain, Hala Tamim

**Affiliations:** 1 College of Medicine, Alfaisal University, Riyadh, Saudi Arabia; 2 Public Health Intelligence, Saudi Public Health Authority, Riyadh, Saudi Arabia; 3 Department of Population Health, King Abdullah International Medical Research Center (KAIMRC), Riyadh, Saudi Arabia; 4 King Saud bin Abdulaziz University for Health Sciences (KSAU-HS), Riyadh, Saudi Arabia; 5 Ministry of National Guard Health Affairs (MNG-HA), Riyadh, Saudi Arabia; 6 Injury Prevention and Control Department, Saudi Public Health Authority, Riyadh, Saudi Arabia; 7 York University, Toronto, ON, Canada

**Keywords:** children, adolescents, severe injuries, Saudi Arabia, ISS

## Abstract

**Introduction:**

Children and adolescents constitute nearly 30% of the global population, and injuries within this age group represent a significant public health concern worldwide.

**Methods:**

A cross-sectional study was conducted across five hospitals within the MNG-HA. The Trauma Registry database extracted all pediatric patients with injuries from 2015 to 2022 who were admitted for at least 1 day to MNG-HA hospitals. The outcome was severe injuries, defined as an Injury Severity Score (ISS) ≥16, and the characteristics of the patients included various demographic, health, and injury-related factors. Bivariate and multivariate logistic regression analyses were reported separately for each age group (0–6, 7–12, and 13–19 years).

**Results:**

Out of 3,382 patients, 16.8% experienced severe injuries, with a higher prevalence among males. Significant associations with severe injuries included drowning, poisoning, and assault in the 0–6 age group; abdominal/spine injuries and motor vehicle crashes, in the 7–12 age group; and poisoning and intentional self-harm in adolescents.

**Conclusion:**

This study identifies critical factors associated with severe injuries across age groups, informing targeted interventions to reduce injury risk in Saudi Arabia.

## Introduction

Injury has become one of the major causes of death and disability among children and adolescents worldwide (1). Patterns of injury in adolescents differ from those in younger age groups, and adolescence represents a developmental transition point for injury risk, owing to factors such as increased independence and tendencies toward risk-taking behaviors (2).

Injuries caused 595,621 deaths and 233,114,563 incident cases worldwide among individuals aged 0–19 years in 2019 (3), claiming more lives than communicable or noncommunicable diseases, nutritional or maternal health causes, or self-harm.

Recent Global Burden of Disease 2019 (GBD 2019) analyses highlighted that unintentional injuries among adolescents are significant contributors to young lives lost and long-term disability (4). For instance, in Turkey, the fatality rate from road traffic injuries among children aged 0–14 years increased from 1.41 per 100,000 in 2006 to 2.13 per 100,000 in 2019, with the highest rate observed among children aged 0–9 years (5). Additionally, falls are the leading cause of injury-related hospitalization in children younger than 5 years old (6). According to the World Health Organization (WHO), violence-related injuries are the leading cause of death among children, with ~0.95 million children and adolescents under 18 dying from injury and violence each year (7). Examining the factors associated with mortality is crucial for comprehending the full impact of injuries.

In Saudi Arabia, injuries were responsible for 82.5% of all preventable deaths among children (8). Studies showed that infants had a higher prevalence of injuries compared to other age groups, and more frequent, severe, and fatal injuries were associated with suspected child abuse (9, 10). Drowning most commonly affected 6–12-year-olds (8). The leading cause of injury-related mortality was road traffic injuries, accounting for 60.6% of these deaths, particularly among 13–18-year-olds (8). Additionally, research from a trauma center in Riyadh found that older patients (>14 years of age) were predominantly male, more likely to be involved in traffic-related injuries, and sustained more severe injuries compared to patients younger than 14 years (11).

In Saudi Arabia, where half of the population is under the age of 25, it is crucial to examine injuries as a public threat to the population under 18 years. Previous local studies on injuries had limited scope, often examining specific types of injuries (burns) without a comprehensive exploration of the range of injuries (12). Furthermore, due to methodological challenges, studies often focus on a single hospital's experience, hindering the exploration of geographical variation in the pattern of injuries (13). The current study attempts to address this gap in the literature to facilitate a more comprehensive examination of injuries in Saudi Arabia. Specifically, the study aimed to (1) describe the characteristics of injuries from all causes among children and adolescents admitted to one of the sub-national health systems in Saudi Arabia and (2) explore factors associated with severe injuries among the same population.

## Materials and methods

### Study design

This was a cross-sectional analytical study utilizing data from the Ministry of National Guard-Health Affairs (MNG-HA) Trauma Registry. The MNG-HA is a nationwide healthcare system in Saudi Arabia that includes five tertiary hospitals with a total bed capacity of 3,737 supported by a network of primary healthcare centers. The system provides medical care to military personnel, their dependents, and civilian members, including students and healthcare workers. The MNG-HA facility in Riyadh also serves as a major tertiary referral center for trauma, receiving critical injury cases requiring specialized emergency care.

### Study population and sample size

The study population consisted of pediatric patients aged 0–19 years who were included in the MNG-HA Trauma Registry between the years 2015 and 2022. The registry includes all patients who sustained an injury and were admitted to an MNG-HA hospital for at least 1 day. From this registry.

### Inclusion and exclusion criteria

Eligible participants included individuals aged 0–19 years who sustained an injury and were hospitalized for at least 24 h at any MNG-HA facility between 2015 and 2022. Patients who were treated and discharged directly from the emergency department, as well as those who died in the emergency room before admission, were excluded from the study. Additionally, cases with incomplete or missing data for key variables required for analysis were not included.

### Variables

The study examined both injury-related and demographic variables. Injury-related variables included the year of injury, location where the injury occurred (categorized as home, street, or other), mechanism of injury (including motor vehicle crashes, falls, burns, drowning, poisoning or self-harm, and other mechanisms), mode of transport to the emergency department (ambulance, private car, or other), and the specific body region affected (head, upper or lower extremities, abdomen, spine, or neck). The extent of injury was also categorized as affecting a single or multiple body regions.

The primary outcome variable was the Injury Severity Score (ISS), a standardized and widely accepted score that quantifies trauma severity by summing the squares of the three highest Abbreviated Injury Scale (AIS) scores from different body regions. The ISS ranges from 1 to 75, and in this study, a score greater than or equal to 16 was used to define severe injury.

Demographic and health-related variables included the patient's age at the time of injury, sex, the geographical region within Saudi Arabia where the hospital admission occurred (Central, Western, or other regions which included Eastern and Northern regions), and the presence of comorbidities such as diabetes, hypertension, smoking, obesity, asthma, anemia, epilepsy, or congenital anomalies. Comorbidities were coded as either present or absent.

Potential confounders were identified and included based on clinical relevance and previous studies. This included age, sex, mechanism of injury, and comorbidities, which were all obtained from the trauma registry using standard definitions. Sex, mechanism of injury, and comorbidities were coded as categorical variables.

### Statistical analysis

Participants were grouped into three age categories: young children (0–6 years), children (7–12 years), and adolescents (13–19 years), to allow for stratified analysis of injury patterns and severity. Descriptive statistics were calculated to summarize the characteristics of the study population within each age group. Bivariate analyses were conducted to assess differences in injury characteristics across age categories.

Multivariable logistic regression models were used to examine the association between the independent variables and the likelihood of sustaining a severe injury, defined as ISS ≥ 16. All relevant covariates were included in the model, and the results were presented as odds ratios with 95% confidence intervals. Statistical significance was set at a *p*-value of < 0.05. All statistical analyses were performed using Stata version 18.5 (StataCorp, College Station, TX, USA).

### Ethical considerations

Ethical approval for the study was obtained from the Institutional Review Board at King Abdullah International Medical Research Center on December 11, 2023 (IRB number 3103/23). The study relied on secondary data that had been fully anonymized prior to analysis to ensure participant confidentiality. Access to the trauma registry was restricted to authorized personnel in accordance with institutional data governance protocols.

## Results

Table 1 shows the descriptive characteristics of the study participants by age group. Injuries were more prevalent among males, notably among 13–19 years of age (83.4%, *p*-value < 0.001). The central region reported the highest proportion of injuries, with 60.5, 58.1, and 65.5% reported for those 0–6, 7–12, and 13–19 years of age, respectively. For the transportation mode, almost half of all injuries were transported to the emergency room via private cars (48%). Adolescents (13–19), compared to younger individuals, reported the highest transportation (28.2%) by police/ambulance (*p* < 0.01). There was a statistical difference between single and multiple body injuries; the older age group (13–19 years) had the highest proportion of having multiple injuries compared to the other two age groups (*p*-value < 0.001). Injuries to the extremities were the most predominant injury type compared to other body region parts. Injuries at home were most prevalent among the age group 0–6 years (66.7%; *p* < 0.001).

**Table 1:** Characteristics of children and adolescents injury patients (*N* = 3,382).

**Variables**	**Age group**				
	**0–6 years**	**7–12 years**	**13–19 years**	**Total**	***p*** **-Value** ^*^
*N*	1,747 (51.7%)	716 (21.2%)	919 (27.2%)	3,382 (100.0%)	
**Demographic and pre-existing health conditions**					
Age at event [mean (SD)]	2.8 (1.8)	9.2 (1.7)	16.2 (1.9)	7.8 (5.9)	<0.001
**Sex**					
Male	1,008 (57.7%)	494 (69.0%)	766 (83.4%)	2,268 (67.1%)	<0.001
Female	738 (42.3%)	222 (31.0%)	153 (16.6%)	1,113 (32.9%)	
**Region in Saudi Arabia**					
Central	1,028 (60.6%)	400 (58.1%)	574 (65.5%)	2,002 (61.4%)	0.001
Western	420 (24.8%)	154 (22.4%)	182 (20.8%)	756 (23.2%)	
Others^a^	247 (14.6%)	135 (19.6%)	121 (13.8%)	503 (15.4%)	
**Comorbidities** ^b^					
Yes	164 (9.4%)	108 (15.1%)	145 (15.8%)	417 (12.4%)	<0.001
No	1,578 (90.6%)	607 (84.9%)	770 (84.2%)	2,955 (87.6%)	
**Injury-related factors**					
**Injury year**					
2015–2019	728 (41.8%)	280 (39.5%)	368 (40.2%)	1,376 (40.9%)	0.528
2020–2022	1,015 (58.2%)	428 (60.5%)	548 (59.8%)	1,991 (59.1%)	
**Injury place**					
Home	1,165 (66.7%)	288 (40.2%)	207 (22.5%)	1,660 (49.1%)	<0.001
Street	121 (6.9%)	137 (19.1%)	377 (41.0%)	635 (18.8%)	
Other^c^	460 (26.3%)	291 (40.6%)	335 (36.5%)	1,086 (32.1%)	
**Mechanism of injury**					
Falls	708 (40.8%)	326 (45.8%)	287 (31.3%)	1,321 (39.3%)	<0.001
MVCs^**^	151 (8.7%)	177 (24.9%)	425 (46.3%)	753 (22.4%)	
Knife/sharp wounds	132 (7.6%)	40 (5.6%)	38 (4.1%)	210 (6.2%)	
Burns	172 (9.9%)	21 (2.9%)	16 (1.7%)	209 (6.2%)	
Drowning	43 (2.5%)	6 (0.8%)	0 (0.0%)	49 (1.5%)	
Intentional self-harm	4 (0.2%)	1 (0.1%)	19 (2.1%)	24 (0.7%)	
Assault	13 (0.7%)	9 (1.3%)	35 (3.8%)	57 (1.7%)	
Poisoning	105 (6.1%)	12 (1.7%)	16 (1.7%)	133 (4.0%)	
Foreign body	305 (17.6%)	63 (8.8%)	15 (1.6%)	383 (11.4%)	
Unknown	101 (5.8%)	57 (8.0%)	66 (7.2%)	224 (6.7%)	
**Transportation mode to hospital**					
Private vehicle	943 (54.1%)	339 (47.3%)	364 (39.7%)	1,646 (48.8%)	<0.001
Ambulance/police	127 (7.3%)	92 (12.8%)	258 (28.2%)	477 (14.1%)	
Unknown	674 (38.6%)	285 (39.8%)	294 (32.1%)	1,253 (37.1%)	
**Body region**					
Single body region	1,596 (91.4%)	591 (82.5%)	643 (70.0%)	2,830 (83.7%)	<0.001
Multiple body regions	151 (8.6%)	125 (17.5%)	276 (30.0%)	552 (16.3%)	
**Injury to extremities**					
No	1,092 (62.5%)	298 (41.6%)	316 (34.4%)	1,706 (50.4%)	<0.001
Yes	655 (37.5%)	418 (58.4%)	603 (65.6%)	1,676 (49.6%)	
**Injury to abdomen/spine**					
No	1,672 (95.7%)	650 (90.8%)	746 (81.2%)	3,068 (90.7%)	<0.001
Yes	75 (4.3%)	66 (9.2%)	173 (18.8%)	314 (9.3%)	
**Injury to head/neck/thorax**					
No	1,356 (77.6%)	552 (77.1%)	656 (71.4%)	2,564 (75.8%)	0.001
Yes	391 (22.4%)	164 (22.9%)	263 (28.6%)	818 (24.2%)	
**Other trauma/external**					
No	1,441 (82.5%)	644 (89.9%)	843 (91.7%)	2,928 (86.6%)	<0.001
Yes	306 (17.5%)	72 (10.1%)	76 (8.3%)	454 (13.4%)	

^*^Chi-squared and ANOVA, ^**^Motor vehicle crashes.

^a^Eastern and Northern regions.

^b^Individuals had one of the following conditions: diabetes, hypertension, smoking, obesity, asthma, anemia, epilepsy, or congenital anomalies.

^c^Recreation places, deserts, public buildings, Residential Institutions, or unspecified.

Table 2 explores the crude and adjusted ORs of severe injury characteristics for the age group 0–19.

**Table 2:** Crude and adjusted multivariate logistic regression for the correlates of injuries, stratified by age groups.

**Covariates**	**Age group**					
	**0–6**		**7–12**		**13–19**	
	**Crude OR (95% CI)**	**Adjusted OR (95% CI)** ^*^	**Crude OR (95% CI)**	**Adjusted OR (95% CI)** ^*^	**Crude OR (95% CI)**	**Adjusted OR (95% CI)** ^*^
**Gender (ref** = **male)**						
Female	1.09 (0.85–1.40)	1.04 (0.77–1.40)	1.27 (0.80–2.01)	1.85 (0.98–3.53)	1.12 (0.72–1.72)	1.22 (0.62–2.37)
**Regions (ref** = **central)**						
Western	0.92 (0.68–1.25)	0.96 (0.67–1.38)	0.58 (0.31–1.09)	0.75 (0.33–1.73)	1.19 (0.79–1.81)	1.07 (0.62–1.86)
Others^a^	1.21 (0.85–1.72)	1.35 (0.89–2.06)	0.97 (0.54–1.71)	2.52 (1.14–5.55)	0.84 (0.49–1.44)	0.93 (0.46–1.89)
**Co-morbidities**^b^ **(ref** = **yes)**						
No	1.06 (0.69–1.64)	1.36 (0.81–2.29)	0.97 (0.54–1.71)	1.31 (0.57–3.03)	0.89 (0.57–1.39)	1.04 (0.56–1.90)
**Injury-related factors**						
**Transportation mode (ref** = **private vehicle)**						
Ambulance/police	4.10 (2.77–6.08)	3.82 (2.23–6.55)	6.17 (3.54–10.74)	3.81 (1.66–8.74)	7.96 (5.13–12.34)	3.69 (1.97–6.90)
Unknown	0.95 (0.72–1.25)	1.74 (1.25–2.43)	0.80 (0.46–1.42)	0.69 (0.33–1.47)	1.05 (0.61–1.79)	1.07 (0.55–2.08)
**Body region (ref** = **one body region)**						
Multiple regions	2.69 (1.87–3.87)	5.46 (2.88–10.38)	6.07 (3.78–9.75)	4.00 (1.45–11.09)	8.56 (5.93–12.38)	2.37 (1.12–4.99)
**Region injured**						
Extremities	0.18 (0.12–0.26)	0.23 (0.13–0.41)	0.34 (0.22–0.54)	0.44 (0.18–1.06)	0.45 (0.32–0.63)	0.62 (0.36–1.07)
Abdomen/spine	3.00 (1.85–4.87)	1.21 (0.67–2.19)	4.65 (2.64–8.20)	3.21 (1.32–7.76)	5.24 (3.63–7.58)	2.68 (1.55–4.62)
Head/neck/thorax	1.43 (1.08–1.90)	0.78 (0.48–1.25)	6.28 (3.94–10.00)	3.43 (1.32–8.91)	11.47 (7.84–16.79)	6.29 (3.40–11.63)
Other^c^	0.67 (0.47–0.95)	0.57 (0.31–1.07)	0.73 (0.32–1.64)	0.44 (0.13–1.54)	1.38 (0.79–2.40)	1.83 (0.80–4.19)
**Injury place (ref** = **home)**						
Street	2.03 (1.34–3.07)	0.83 (0.32–2.13)	3.42 (2.02–5.77)	5.10 (1.35–19.32)	2.53 (1.62–3.94)	0.72 (0.27–1.91)
Others^d^	0.68 (0.50–0.92)	0.74 (0.50–1.09)	0.58 (0.32–1.05)	1.29 (0.56–3.00)	0.58 (0.34–0.99)	0.66 (0.30–1.45)
**Mechanism of injury (ref** = **fall)**						
MVC^*^	4.70 (2.98–7.42)	1.83 (0.73–4.61)	6.51 (3.43–12.35)	0.51 (0.14–1.83)	9.44 (5.11–17.46)	1.33 (0.47–3.81)
Knives/sharp wounds	0.10 (0.01–0.70)	0.15 (0.02–1.13)	–	–	0.62 (0.08–4.90)	0.75 (0.09–6.62)
Burns	0.86 (0.44–1.69)	0.57 (0.22–1.51)	2.35 (0.50–11.08)	5.49 (0.68–44.39)	3.27 (0.67–16.06)	0.53 (0.03–8.84)
Drowning	9.99 (5.14–19.42)	6.45 (2.70–15.40)	–	–	–	–
Poisoning	9.46 (5.86–15.27)	6.26 (3.37–11.64)	11.14 (2.99–41.47)	18.84 (3.63–97.76)	17.82 (5.68–55.98)	11.89 (3.15–44.90)
Assault	5.61 (1.67–18.82)	4.00 (1.11–14.40)	6.37 (1.21–33.49)	2.12 (0.30–15.10)	3.82 (1.26–11.58)	0.78 (0.17–3.61)
Foreign body	7.74 (5.38–11.15)	5.34 (3.20–8.89)	13.71 (6.55–28.70)	22.40 (7.73–64.89)	11.46 (3.39–38.78)	14.65 (3.58–59.96)
Intentional self-harm	–	–	–	–	13.37 (4.46–40.03)	11.89 (3.15–44.90)
Unknown	1.94 (1.03–3.64)	1.27 (0.59–2.71)	0.44 (0.10–1.96)	0.66 (0.13–3.41)	4.09 (1.69–9.94)	2.98 (0.93–9.55)

^a^Eastern and Northern regions.

^b^Comorbidities: (diabetes meletus, epilepsy, depression, congenital anomalies, dyslipidemia, asthma, and anemia).

^c^External injuries and suffocations.

^d^Recreation places, desert, Public Buildings, Residential Institutions, and unspecified.

^*^Motor Vehicle crashes.

### Age 0–6 years

Children transported by ambulance or police had higher odds of having experienced severe injuries compared to those transported by private vehicles (aOR: 3.82, 95% CI: 2.23–6.55). Injuries involving multiple body regions were associated with increased severity (aOR: 5.46, 95% CI: 2.88–10.38), while injuries to the extremities were linked to lower severity (aOR: 0.23, 95% CI: 0.13–0.41). Mechanisms such as drowning (aOR: 6.45, 95% CI: 2.70–15.40), assault (aOR: 4.00, 95% CI: 1.11–14.40), poisoning (aOR: 6.26, 95% CI: 3.37–11.64), and foreign bodies (aOR: 5.34, 95% CI: 3.20–8.89) significantly elevated the odds of severe injuries compared to falls.

### Age 7–12 years

In this group, being transported by ambulance or police was significantly associated with severe injuries (aOR: 3.81, 95% CI: 1.66–8.74). Injuries involving multiple body regions (aOR: 4.00, 95% CI: 1.45–11.09), as well as injuries to the abdomen/spine (aOR: 3.21, 95% CI: 1.32–7.76) and head/neck/thorax regions (aOR: 3.43, 95% CI: 1.32–8.91), were linked to higher injury severity. Sustaining injuries on the street (aOR: 5.10, 95% CI: 1.35–19.32) and mechanisms like poisoning (aOR: 18.84, 95% CI: 3.63–97.76) and foreign bodies (aOR: 22.40, 95% CI: 7.73–64.89) were also associated with increased severity.

### Age 13–19 years

Adolescents transported by ambulance or police had higher odds of severe injuries (aOR: 3.69, 95% CI: 1.97–6.90). Injuries affecting multiple body regions (aOR: 2.37, 95% CI: 1.12–4.99), the abdomen/spine (aOR: 2.68, 95% CI: 1.55–4.62), and head/neck/thorax (aOR: 6.29, 95% CI: 3.40–11.63) were linked to greater severity. Intentional self-harm (aOR: 11.89, 95% CI: 3.15–44.90), poisoning (aOR: 11.89, 95% CI: 3.15–44.90), and foreign bodies (aOR: 14.65, 95% CI: 3.58–59.96) were significant mechanisms associated with severe injuries.

These findings underscore the importance of transport mode, injury location, affected body regions, and injury mechanisms in determining injury severity across different pediatric age groups.

## Discussion

This study examined the patterns and determinants of severe injuries among children and adolescent patients (0–19 years) admitted to the Ministry of National Guard-Health Affairs (MNG-HA) hospitals in Saudi Arabia between 2015 and 2022. The findings reveal significant age-related differences, with younger children accounting for half of the sample and primarily sustaining injuries at home and being transported by private vehicles, while adolescents more often experienced multiple injuries and were transported by ambulance. This study excluded both patient with trauma who were treated and discharged directly from the emergency department (potentially indicating very minor injuries), and patients who died before hospital admission. This exclusion may underestimate the overall burden as well as the true spectrum of pediatric trauma in this source population. However, as the vast majority of individuals with any trauma are often admitted at least for 24 h for observation following an injury, the impact of this exclusion is expected to be minimal.

The exclusion of those who were dead on arrival or died at the emergency room was due to the design and eligibility criteria of the trauma registry itself (i.e., including those with any trauma requiring admission). This exclusion, indeed, masks our ability to examine fatal injuries among pediatric population, and our estimates can be considered as a lower bound of the trauma burden within the pediatric population in Saudi Arabia. We encourage future researchers to examine the availability of data from alternative resources (e.g., Red Crescent or other similar organizations) that are beyond the scope of the Trauma registry.

Studies have consistently found that younger children are particularly vulnerable to unintentional injuries within the home environment. This is explained by their developmental drive to explore, coupled with limited capacity to recognize potential hazards (14). This heightened susceptibility places them at increased risk of sustaining injuries that may lead to long-term disability or death (14). Additionally, specific injury mechanisms, such as poisoning, self-harm, and foreign bodies, were strongly associated with severe trauma across age groups. These insights contribute to a better understanding of the severity of injuries among children and adolescent and highlight the need for age-specific injury prevention strategies, enhanced parental supervision, and targeted public health interventions to reduce injury risk and improve outcomes.

The study revealed that males were more likely to sustain injuries requiring hospital admission compared to females (67 % vs. 33%). This finding aligns with previous studies from Saudi Arabia's Eastern region and elsewhere reporting more injuries among males than females (15, 16). This gender-based disparity in the prevalence of injuries, as demonstrated by multiple studies, may be attributed not only to boys' greater tendency to engage in risk-taking behaviors compared to girls, but also to parental influences (17). A growing body of research suggests that parents contribute to these sex differences by engaging in gender-differentiated socialization, which shapes children's understanding of gender identity and influences their behavior in risk-related situations. In this case, public health interventions should be specifically designed to address the diverse and intersecting factors that influence injury risk, such as gender, race, socioeconomic status, disability, and environmental context. These interventions may include targeted counseling, culturally responsive safety education, community-based outreach programs, and inclusive training initiatives that empower youth with the knowledge and skills to reduce their injury risk. The Transforming Injury Prevention for Youth (TrIPY) model exemplifies this approach by applying an intersectional lens to developing and implementing injury prevention strategies, ensuring they are both equitable and effective across diverse populations (18).

When examining transportation methods, ambulance or police transport was the most used in the older age group (13–19), accounting for 28.2% of cases. This aligns with a study in the USA, which found that children aged 12–18 years were more likely to use ambulance transport (15). However, a Canadian study reported a lower average age for ambulance transport usage, with a mean age of 9.65 years (16). These variations may arise from various countries' injury mechanisms and transportation policies. Transport by ambulance or police was significantly associated with severe injuries across all age groups. This is consistent with an Australian study that found severely injured children were more likely to be transported by ambulances (19). These results also align with local data where Alghnam et al. (20) showed that patients transported by ambulance may have more severe injuries. Conversely, Rominger et al. (15) reported that ambulances might sometimes be used for non-critical transport, while critically ill children arrive to the ED by other means.

In terms of body regions affected, the older age group (13–19) reported the highest incidence of multiple body injuries (30.0%). This is consistent with findings from the USA, which occurred in the 13–19 age group (21). Additionally, the same age group had the highest proportion of abdomen/spine injuries (18.8%). This is similar to a study in Taiwan that reported a higher incidence of severe abdominal injuries in older children compared to younger ones (22). In contrast, a Nigerian study found that 31.4% of children under 15 years of age had a mean age of 2.30 years for abdominal injuries (23). Studies have suggested that the increase of this type of injury in this age group is often related to Motor Vehicle Accidents (MVA) (21).

In our results, Motor vehicle collisions (MVCs) were more frequent in the older age group (46.3%). This is consistent with a Spanish retrospective study, where the mean age for MVCs was 14.4 years (median: 16) (24). However, another study showed an increase in falls and road traffic accidents between 0–2 and 3–6 years, with a subsequent decrease between the ages of 7–10 (25). The higher prevalence of MVCs in older children could be attributed to increased road traffic exposure as they gain more independence in mobility (23), combined with ongoing brain development, affecting impulse control and risk assessment abilities, contributing to risky behaviors such as unsafe driving (26). These behaviors include speeding, distracted driving, and failure to use a seatbelt. Programs like Graduated Driver Licensing (GDL), implemented in the United States, have been found to reduce crash risk and promote safer driving habits with 19% reduction for injury crashes and about 21% reduction for fatal crashes for 16-year-olds. GDL is a structured programs that phase in driving privileges for new drivers over time through a series of stages, typically including a supervised learner period, an intermediate license with restrictions, and full licensure (27).

Regarding the mechanism of injury, falls, burns, poisoning, and foreign bodies were more common in the 0–6 age group, with respective rates of 40.8, 9.9, 6.1, and 17.6%. This aligns with a cohort study from England, which found that burns in children aged 0–4 were most often caused by exposure to heat, such as hot drinks or bathwater, accounting for 74.2% of burns in this age group (28). This is attributed to increased curiosity in this age group and the frequent presence of potentially hazardous household items. Additionally, a US study found that foreign body injuries most frequently occurred in children aged 1 year (21.3%) (29). Conversely, Baker et al. (28) noted that individuals aged 15–24 had higher rates of poisoning, which could be explained by an increase in substance misuse and mental health challenges that often lead to overdoses. Falls were also found to be common in the 6–11 age group, occurring in 32% of children in a Swiss study (30), with another study by Hyder et al. (24) reporting that an average of 36% of all injuries in children under 5 years old were due to falls, with Africa showing an incidence rate of 786 per 1,000.

Intentional self-harm and assault were also more prevalent in the 13–19 age group (5.9%), aligning with findings from an English study, which observed the highest prevalence among 16- and 17-year-olds (29). As Cunningham suggested, violence may explain this trend. Griffen et al. also found that the rate of self-harm among individuals aged 10–24 was 318 per 100,000, with peak rates in females aged 15–19 years (564 per 100,000) (31). Another study conducted in England also found a higher incidence of self-harm among females than males (31). Adolescent self-harm often has a significant impact both individual and families, contributing to stress, anxiety, and financial strain (31).

Multiple body injuries were also significantly associated with severe injury in all age groups: 0–6, 7–12, and 13–19 years. This is consistent with findings in the USA, where most multiple injuries occurred in the 13–19 age group (21).

Foreign body injuries were significantly associated with severe injuries across the age groups 0–6, 7–12, and 13–19. Complications from foreign bodies range from mucosal abrasions to death if they remain impacted for extended periods (32, 33). A retrospective study also found that coins were the most commonly ingested foreign body and that children who ingested coins were 2.43 times more likely to be hospitalized (29), which aligns with findings that foreign body-related injuries are a significant concern among adolescents aged 15–19 (34).

In the 7–12 and 13–19 age groups, abdomen/spine injuries were significantly associated with severe injuries. These findings are consistent with a study conducted in India, which reported higher odds of severe abdominal/spine injuries in children under 13 (34). A similar trend was observed in the USA, where spine injuries were more severe in older children (35).

Head/neck/thorax injuries were also significantly associated with severe injuries in the 7–12 and 13–19 age groups. This is similar to findings in the USA, where thoracic injuries were more prevalent in older patients (35). And to data from the National Trauma Data Bank, which identified head injuries as a major cause of in-hospital pediatric mortality and could be correlated with increased MVC injuries in this age group (36).

### Study strengths and limitations

The study has several strengths. First, to the best of our knowledge, this is the first multi-center study to document the characteristics of severe injuries among children and adolescents in Saudi Arabia. Second, the registry includes data collected from five tertiary care centers across Saudi Arabia, increasing the representativeness of the current study. Further, the center in Riyadh is one of only two level III trauma centers in Saudi Arabia and accepts referrals from all over the region further enhancing the external validity of the results. Third, this study included a wide age range (0–19), which enabled examining different patterns of trauma that evolve with chronological age and facilitated identifying priorities for researchers and policymakers. Lastly, although the study is cross-sectional, the nature of the exposure (any injury) and outcome (injury severity score) establishes a natural temporality in this study that are seldom available with cross-sectional studies.

On the other hand, the results discussed in this study should be interpreted considering a few limitations. First, although the results are collated from several areas within Saudi Arabia, it will miss individuals who received care elsewhere. Second, the data within the trauma registry depend, as other registries, on the quality of documentation by healthcare providers. However, as there were periodic checks on the trauma-related variables, we hope that the potential misclassification is mitigated. Additionally, long-term outcomes could not be examined because the registry included data up until the discharge date. Third, data on important variables such as seatbelt use, time from injury to hospital admission, and specific poisonous agents were unavailable, limiting our ability to examine their association with the severity of the injury. Lastly, patients who were discharged without admission or died in the emergency room were excluded, which can lead to an underestimation if the true severity and burden of pediatric trauma as discussed earlier.

## Conclusion

The study revealed critical associations between severe injuries and specific factors across different pediatric age groups. For children aged 0–6 years, drowning, poisoning, and assault were significantly linked to severe outcomes. In the 7–12 age group, injuries to the abdomen/spine, incidents occurring on the street, and poisoning emerged as key factors. Among adolescents aged 13–19 years, poisoning and intentional self-harm were highly associated with severe injuries. These findings highlight the urgent need for targeted interventions tailored to the unique risks faced by each age group. As injury remains a significant public health concern, these insights can inform prevention strategies and guide future population-based research across Saudi Arabia. Additionally, the recent World and European reports on child injury prevention provide accessible evidence and effective approaches to address the major types of injuries, drowning, falls, poisoning, burns, and traffic-related incidents, that contribute to injury-related morbidity and mortality.

## Data Availability

The datasets presented in this article are not readily available because the dataset is governed by a Saudi entity, and I am not authorized to publish it or make it available upon request. Requests to access the datasets should be directed to ResearchOffice@mngha.med.sa.
