# Supplementary material for: Characteristics of severe injuries among children and adolescents in a sub-national trauma registry in Saudi Arabia
Source: Front Public Health. 2025 Nov 27;13:1651372. doi: 10.3389/fpubh.2025.1651372 (PMC12696180; doi:10.3389/fpubh.2025.1651372)
Supplement: Supplementary file 1 [file Table_1.docx]

Supplementary: Table of Comorbidities

| Comorbidities | Frequency |
| --- | --- |
| behavioral smoker | 19 |
| cardio hypertension | 6 |
| congenital anomalies | 8 |
| endocrine diabetes | 18 |
| endocrine obesity | 34 |
| endocrine dyslipidemia | 2 |
| functional dependent | 2 |
| hematoma anemia | 6 |
| Hematoma bleeding | 0 |
| malignancy cancer | 6 |
| mental depression | 1 |
| Neuro epilepsy | 23 |
| Renal | 1 |
| Asthma | 87 |
| Respiratory disease | 2 |
| * Other comorbidity | 258 |
| **Mutli-Comorbidties** |  |
| 1 Comorbidity | 369 |
| 2 +Comorbidities | 47 |
|  | |

* ADHD, Autism, developmental delay, Eczema, Allergies, Down syndrome, Sickle cell, Thalassemia, Anxiety, and Psychosis
